# Age-Induced Loss of Mossy Fibre Synapses on CA3 Thorns in the CA3 Stratum Lucidum

**DOI:** 10.1155/2013/839535

**Published:** 2013-06-24

**Authors:** Bunmi Ojo, Heather Davies, Payam Rezaie, Paul Gabbott, Francis Colyer, Igor Kraev, Michael G. Stewart

**Affiliations:** Department of Life, Health and Chemical Sciences, The Open University, Walton Hall, Milton Keynes MK7 6AA, UK

## Abstract

Advanced ageing is associated with hippocampal deterioration and mild cognitive decline. The hippocampal subregion CA3 stratum lucidum (CA3-SL) receives neuronal inputs from the giant mossy fibre boutons of the dentate gyrus, but relatively little is known about the integrity of this synaptic connection with ageing. Using serial electron microscopy and unbiased stereology, we examined age-related changes in mossy fibre synapses on CA3 thorny excrescences within the CA3-SL of young adults (4-month-old), middle-aged (12-month-old), and old-aged (28-month-old) Wistar rats. Our data show that while there is an increase in CA3 volume with ageing, there is a significant (40–45%) reduction in synaptic density within the CA3-SL of 12- and 28-month-old animals compared with 4-month-old animals. We also present preliminary data showing that the CA3 neuropil in advanced ageing was conspicuously full of lipofuscin and phagolysosome positive, activated microglial cellular processes, and altered perivascular pathology. These data suggest that synaptic density in the CA3-SL is significantly impaired in ageing, accompanied by underlying prominent ultrastructural glial and microvascular changes.

## 1. Introduction

Age-related hippocampal deterioration and deficits in cognitive function (spatial memory) [1, 2] are correlated with ultrastructural changes in the CA3 hippocampal subregion, including (i) loss of synapses and integral synaptic proteins (CA3-SR) [3–11], (ii) significant microglia activation [12], (iii) changes in synaptic plasticity [13], and (iv) altered synaptic-glial interactions [9]. The functional integrity of mossy fibre axons whose large giant boutons project to the dendritic spines (thorny excrescences) located in the CA3 stratum lucidum (SL) is essential in both long-term potentiation (LTP) and storage and recall of spatial representation on modifiable synapses of recurrent collaterals of the CA3 pyramidal cells [14]. Electron microscopic studies have reported marked ultrastructural plasticity of synaptic connections in the CA3-SL, in correlation with neurobehavioral performance [4]. Although a large body of data has reported significant age-related ultrastructural changes in the CA3, very little is currently known about the integrity of these mossy fibre synaptic connections in the CA3-SL with advanced ageing. Our aim was to use unbiased stereological methodology to determine whether subtle age-related morphological changes could be demonstrated in the relatively unexplored mossy fibre synapses which form on CA3 thorny excrescences in Wistar rats. The right dorsal hippocampus was examined, because the dorsal hippocampal formation appears to be more specialised than the ventral formation for memory and spatial learning [15] and thus has been the focus of our previous investigations. We demonstrate an increase in CA3 volume with ageing but a significant reduction in mossy fibre synapse (density) on CA3 thorny excrescence in the CA3-SL with ageing. We also observed and present our preliminary findings showing that the CA3 neuropil in advanced ageing was conspicuously full of lipofuscin and phagolysosome positive, activated microglial cellular process, and altered perivascular cell morphology.

## 2. Materials and Methods

Animals male Wistar rats aged 4 months (250–350 g), 12 months, or 28 months (400–550 g) supplied from Harlan UK were housed in pairs with a 12 h light schedule at ambient temperature. Healthy young adult, middle-aged, and old-aged rats were used in this study (*n* = 3). Experiments were performed under a license issued by UK home office and in accordance with the guidelines laid down by the local ethical committee at the Open University. Tissue preparation and processing for light microscopy animals were deeply anesthetised with urethane (1.5 g/kg), perfused transcardially with 100 mL of physiological saline, followed by 100 mL of 3.5% paraformaldehyde and 3.75% acrolein in 0.1 M phosphate buffer (pH 7.4) at room temperature. After perfusion, the brains were removed from the skull, postfixed and placed in 0.1 M phosphate buffer solution, and processed as described previously [13]. A consecutive series of 50 *μ*m thick coronal sections were cut using the Leica VT100 vibrating microtome, throughout the extent of the whole hippocampus. Sections were stored in −20°C in 30% sucrose and 30% ethylene glycol in PB.

### 2.1. Light Microscopy: Volume Estimation

**Figure 1:**
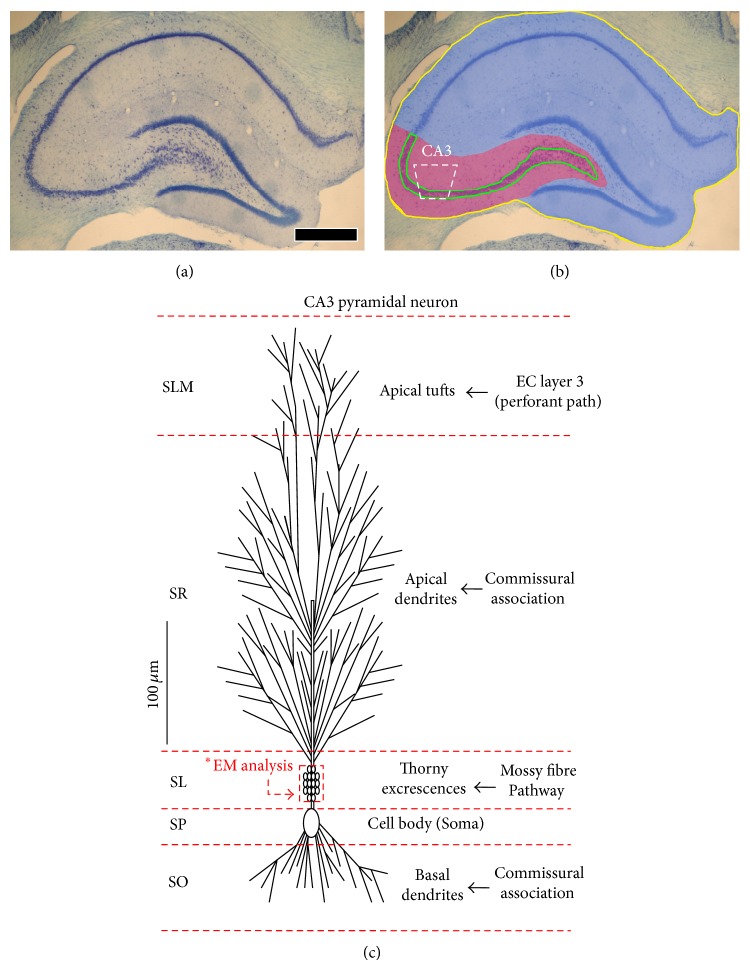
Boundaries of the CA3 and the right dorsal anterior hippocampus used for volumetric analysis. Top panel images show a Nissl stained section from the right dorsal anterior hippocampus (a, b) with the CA3 subfield (purple and cell layer in green) and the right dorsal hippocampi (blue/yellow outline) demarcated in distinct colouring shown in (b). EM analysis and sections were obtained from the CA3 subregion. Bottom panel shows a CA3 pyramidal neuron orientation as described by Spruston [19] and its distinct cellular layers. The CA3 receives three main inputs from (i) entorhinal cortex (EC) layer 3 forming the perforant pathway, (ii) recurrent collateral CA3 commissural fibres, and (iii) the *giant mossy fibre boutons* from the dentate gyrus. The area demarcated in red in the CA3 stratum lucidum (SL) was chosen for EM analysis. Abbreviation: stratum radiatum (SR), stratum oriens, stratum pyramidal layer (SP). Scale bar represents 540 *μ*m in (a) and (b).

The volume of the right dorsal anterior hippocampus was determined using the Cavalieri method [16] as described elsewhere in relation to hippocampus (initial boundaries; bregma −1.80 to −4.16) [17, 18]. Stained 1 in 5 serial sections (10 total) through the full extent of the region of interest was viewed at low magnification using a Nikon E600 digital photomicroscope, and digital images were analysed using the stereo-investigator software package (MicroBrightField Inc., USA) (assessed blind to grouping). For each animal, the total volume of the right dorsal anterior hippocampus and the CA3 subregion (Figures 1(a) and 1(b)) was subsequently derived by multiplying the calculated mean surface area by the section thickness (*t* = 50 *μ*m) and the total actual number of sections (*n*) in which the right dorsal anterior hippocampus occurred.

### 2.2. Electron Microscopy (EM)

Sections for electron microscope analysis were processed as previously described [6, 20]. Briefly, the 50 *μ*m right dorsal anterior hippocampal slices were postfixed in 2% osmium tetroxide diluted in PB for one hour at RT. Sections were dehydrated in graded aqueous solution of acetone and infiltrated with a (50 : 50) solution mixture of 100% acetone and Epon overnight in capped vials at RT and polymerised at 60°C for 48 hours. The embedded slices on the block surface were trimmed to isolate the area of interest. A Leica UCT ultramicrotome was used to obtain ultrathin serial sections of 60–70 nm thickness from the area of interest and collected on formvar-coated copper slot grids. The sections were counterstained with uranyl acetate, followed by Reynolds lead citrate before digital image acquisition via an inline AMTXR60 digital camera on a JEM 1400 transmission electron microscope.

### 2.3. EM Examination and Stereological Analysis of Synaptic Density

**Figure 2:**
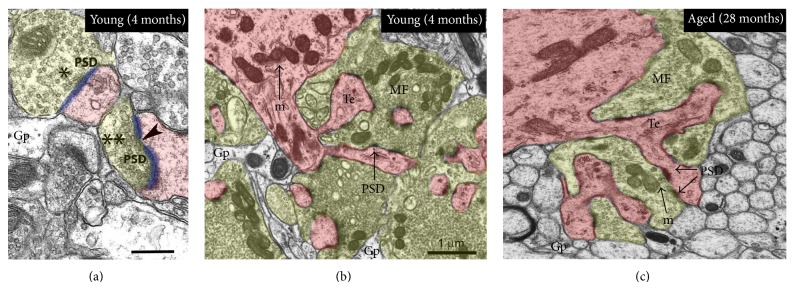
Electron micrographs showing the contrast between CA3 SL tissue from young adult (4 months) and aged (28 months) tissue. Qualitatively, tissue from the young rats and aged rats showed some differences, most notably fewer vesicles present in the mossy fibre boutons of the aged animals, and in the 4-month-old animal mitochondria are more numerous. Single and double asterisks in (a) denote macular and perforated synapses, respectively (based on observation of serial sections). Abbreviation: postsynaptic density (PSD), mitochondria (m), thorny excrescence (Te; pink), mossy fibre (mf; yellow), glial processes (Gp). Scale bar: 1.0 *μ*m in (b)-(c) and 0.2 *μ*m in (a).

The CA3-SL was selected for synaptic density (*N*_*v*_) estimates and neuropil EM examination (Figure 1(b)). The CA3-SL was easily identifiable because it was adjacent to the cell bodies in the pyramidal layer and contained numerous giant mossy fibre boutons (MFBs) with many small synaptic vesicles and dendritic spines (or thorny excrescences; consisted of several thorns with a rounded bulbous shape) (Figures 2(a)–2(c)). Ultrastructural characteristics, defined according to criteria described by [21], were used to identify asymmetric synapses on MFBs, which had thin presynaptic membrane specialization with two or more vesicles and a thick postsynaptic synaptic density (the vast majority of excitatory synapses in this region). Synapses were further classified as macular when the postsynaptic density (PSD) was unperforated and perforated (or segmented) when the PSD had one or more notable discontinuity in the electron density of the postsynaptic junction [13, 22]. Glial processes were also easily distinguishable under EM examination, because they had an irregular shape, in close apposition with neighboring neurons (Figures 2(a) and 2(b)).

The synaptic density (*N*_*v*_) was estimated using the unbiased double disector technique [23]. Digital images were taken using an AMTXR60 digital camera attached to a JEM 1400 transmission electron microscope (at 8000x column magnification). Images were taken only in the CA3-SL, from the proximal part of the apical dendrites where the large giant mossy fibre boutons form synaptic contacts with thorny excrescences (dendritic spines) of the CA3. To calibrate the double dissector, a trial run was performed (using the small fold and relative electron transmission (RET) method) to empirically determine the height of the disector or thickness separation between the pair of examined sections required for the disector technique. In total twenty disector pairs per animals were analysed (at a 1 in 3 series) using an unbiased counting frame (8 × 6 *μ*m). From each disector pair, the total asymmetric synapse number and type of synapse (macular or perforated) were determined. For statistical accuracy a minimum of >100 profiles were counted. The number of synapses present in the reference frames that were not in the look-up frame was recorded (Σ*Q*−). To perform a double disector method, the reference and look-up frames were then interchanged, and the procedure was repeated again. The number of synapses per unit volume (per mm^3^) (*N*_*v*_) was then calculated using the formula *N*_*v*_ = Σ*Q* − /*ah*, where *a* is the area of the unbiased counting frame and *h* is the distance between the two sections [23]. Number of phagolysosome positive microglia process and number of phagolysosome per microglial cell process was determined by 2D morphometric analysis. Glial cells as indicated above were easily distinguishable on EM images; only clearly distinguishable electron dense globule or spherical phagolysosomes vacuoles were counted. Analysis was performed in the CA3-SL on 20 captured images, within a total area of 71.25 *μ*m^2^.

### 2.4. Statistical Analysis

Graphs were prepared using Prism 5.0 software and data analysed using the Statistical Package for the Social Sciences program (SPSS version 17, SPSS Inc., Chicago, USA). One way analysis of variance (ANOVA) was used with criterion *P* < 0.05 to assess group differences, followed by Tukey's unequal *N* honest significant differences test.

## 3. Results 

### 3.1. CA3 Hippocampal Volume Increase (Subtly) with Ageing

The data from young (4-month-old), middle-aged (12-month-old), and old-aged (28-month-old) rats (Table 1) show, that there is a significant age-related increase in the CA3 subregion volume of the right dorsal hippocampus. Both 12- and 28-month-old animals showed a significant and progressive increase along the developmental continuum in CA3 volume estimates compared to young adult (4-month-old) controls (Figure 1(a)). However, when the whole total right dorsal anterior hippocampus was taken into consideration, there was only a significant increase in volume estimates after 28 months of age compared to 4-month-old controls (Figure 1(b)).

### 3.2. Loss of Asymmetrical Synapses in the CA3 Stratum Lucidum with Ageing

Examination of mossy fibre asymmetric synapses on CA3 thorny excrescences, using unbiased stereological methods indicate (Table 1) that there was a trend showing a (40–45%) loss in the numerical density of asymmetrical excitatory synapses within the CA3-SL at both 12 and 28 months compared to young (4-month-old) rats. Macular, perforated, and total mossy fibre synapse density was significantly reduced throughout, at 28 months of age compared to control 4-month-old young adults. At 12 months old, only macular subtype mossy fibre synaptic density was significantly reduced compared to control young (4-month-old) animals. No changes were detected in synaptic density estimates taken between middle-aged (12-months-old) animals compared to old aged (28-month-old) animals. Qualitatively, tissue from the young rats and aged rats also showed some differences in CA3 mossy fibre bouton, most notably fewer vesicles and mitochondria present in the advanced aged (Figures 2(a)–2(c)).

### 3.3. Qualitative and Quantitative Changes in Microglia with Advanced Ageing in the CA3-SL

**Figure 3:**
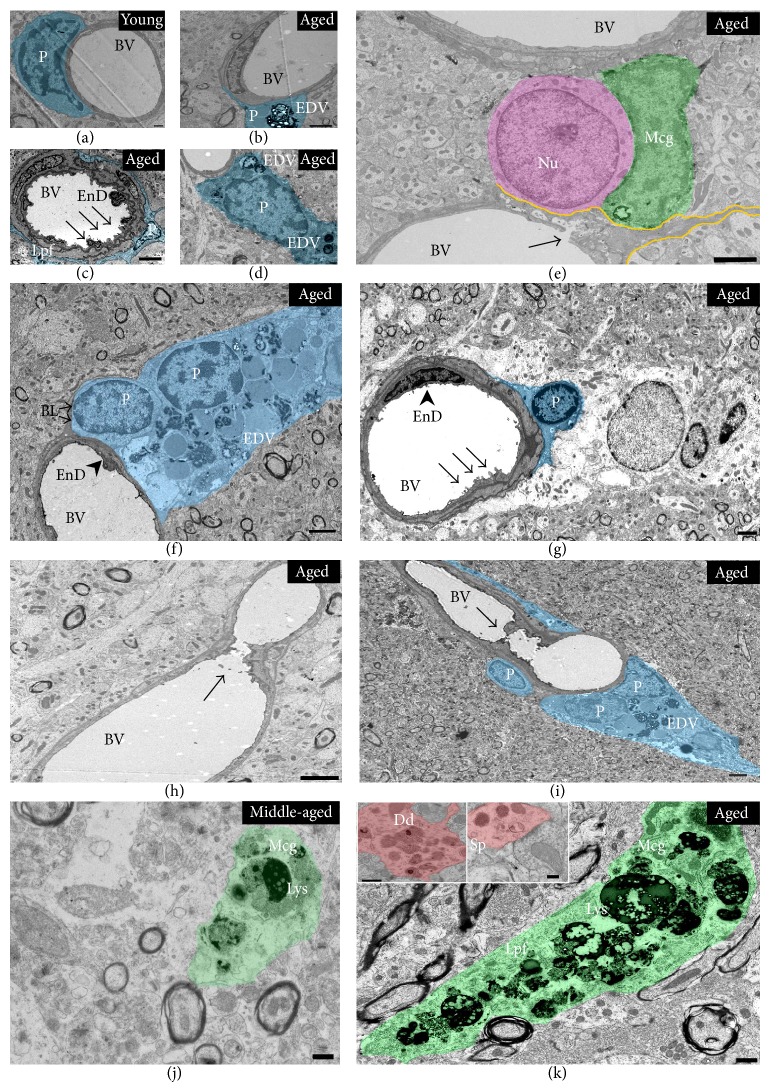
Putative phagocytic activated microglia and altered microvascular pathology with advanced ageing in the CA3. Microvessels in the CA3 region of advanced aged animals (from 22 to 28 months old) were occasionally surrounded by perivascular cells with electron dense vesicles/lipofuscin deposits (b, d, f), compared to healthy microvessel at 4 months (a). Moreover, endothelial cell membrane lining the lumen of vessels also appeared to be structurally irregular and dysmorphic in some cases ((c, g); see arrow), with evidence of shedding of pinocytic vesicles into the lumen ((g); see arrow). There was evidence of what appeared to be leaky microvessel into the brain parenchyma ((e); see yellow demarcated line and arrow showing damage to endothelial cell membranes lining the blood vessel). On very few occasions, atherosclerotic plaques were noticeable in the lumen of a small number of microvessels with advanced ageing ((h, i); see arrow). At 12 months and more prominently at 28 months (j, k), the neuropil of the CA3-SL exhibited numerous conspicuous evidence of potentially activated microglial processes, with a dark cytoplasm, presumably full of lipofuscin deposits and spherical phagolysosomes vacuoles. Some postsynaptic structures in advanced ageing demonstrated electron dense spherical vesicles (see (k) images inset; dendrite and spine in red). Abbreviation: (Mcg) microglia in green, (Lys) phagolysosome, (Lpf) Lipofuscin, (EDV) electron dense vesicles, (P) perivascular cells (blue), (EnD) endothelial cell, (BV) blood vessel, (BL) basal lamina, (Sp) spine, (Dd) dendrite, (Ast) astrocyte, (Nu) neuronal nucleus. Scale bar represents 0.5 *μ*m in (a–d, f, j, and k) and 2 *μ*m in (e and g–i).

Examination of the neuropil of CA3 to identify any age-related ultrastructural changes showed that aged (28 months) animals exhibited conspicuous evidence of activated microglial processes with several electron dense spherical fragments, which presumably resembled phagolysosomes and lipofuscin deposits (Figure 3(k)). In addition, in 12-month-old (middle-aged) animals there was some evidence of activated microglial processes (see Figure 3(j)). However, the content of phagolysosome per microglial cell was 50% greater with advanced age compared to 12-month-old animals (Table 1; see bottom panel).

Advanced aged animals showed alterations to postsynaptic compartments (dendrites and spines) with evidence of large deposits of electron dense spherical structures (Figure 3(k); inset). There was also evidence of occasionally altered perivascular cell morphology with electron dense particles and vesicles, especially at advanced ageing (Figures 3(b), 3(d), 3(f), and 3(g)), dysmorphic microvessels with irregularly shaped/damaged endothelial cells (Figures 3(c), 3(e), and 3(g)), and presumably atherosclerotic plaques in the lumen of microvessels (Figures 3(h) and 3(i)). In contrast, young (4-month-old) animals showed no signs of activated glia processes or altered perivascular integrity, and the neuropil appeared healthy, with fine ultrastructural profiles (Figures 2(b) and 3(a)).

## 4. Discussion

This study has investigated age-related qualitative and quantitative changes in mossy fibre synapses within the CA3-SL region of young adults (4-month-old), middle-aged (12-month-old), and old (28-month-old) Wistar rats. The data presented show a small but significant increase in the volume of the CA3 with ageing, while mossy fibre synapse densities (on CA3 thorns) were significantly reduced (up to 45%) with ageing. Activated microglial processes and altered perivascular cell morphology with electron dense spherical fragments were also conspicuously distributed throughout the neuropil of the CA3 with advanced ageing.

### 4.1. Subtle Increase in CA3 Hippocampal Volume with Ageing

A major challenge in the study of advanced (cognitive) brain ageing in humans is to define significant boundaries of normal physiological changes as distinct from age-related pathological conditions, such as mild cognitive decline (MCI), vascular dementia, or Alzheimer's disease (AD). Volumetric changes in the brain have long been considered as an important marker/indicator of cognitive decline. Imaging studies have been helpful to identify significant changes in brain volume, especially in the “hippocampus.” These volume estimates have been shown to decrease from 0.1-0.2% per year between the age of 30–50 years, rising to 0.5% per year for those who are over the age of 70 years.

Our findings of age-related significant (but subtle) increases in volume of CA3 (and small increases in the hippocampus as a whole) appear to be counterintuitive and in contrast to previous studies which have reported evidence of hippocampal atrophy with advanced ageing. However, our finding is also supported in part by stereological based studies that investigated principal cell fields of the hippocampus (CA1 and CA3 pyramidal cell layer and granule cell layer) in rodents. Those provided evidence only of “small regional changes” in neuronal cell densities/volumes [10, 24], which are indistinguishable from aged rats that exhibit spatial learning/memory deficits in relation to young animals. Rapp and Gallagher [10] thus concluded that “hippocampal neuronal degeneration (or atrophy) is not an inevitable consequence of ageing as pyramidal neurons (*their cell bodies*) are preserved in the rodent hippocampus.” Changes in CA3 volumes with ageing may therefore be attributed to changes in nonneuronal cells such as glial cells (astrocyte, microglia, oligodendrocytes), neurite modification, or alterations to the vasculature.

### 4.2. Loss of Mossy Fibre Synapse (Density) on CA3 Thorns with Ageing

We report age-related reductions in total mossy fibre asymmetric (perforated, macular) synaptic density on CA3 thorny excrescences, 43% at 12 months and 45% at 28 months compared to 4 months. While macular synapse density decreases significantly at both 12 and 28 months, perforated synapse density decreases significantly only at 28 months. Asymmetric mossy fibre synapses are one of the three main glutamatergic excitatory synapses in the hippocampus thought to be important in NMDA receptor dependent and independent LTP (an experimental correlate of learning and memory processing). Although the functional significance of the loss of these asymmetric synapses (and especially the perforated type thought to be involved in the expression of LTP) in the hippocampal microcircuitry is unknown, it could represent one of the significant neurobiological substrates responsible for the deteriorations in hippocampal function with ageing [1]. However, because almost no change in CA3 asymmetric synapse density was observed between 12 and 28 months of age (implying that these synapses are relatively stable from middle adulthood into old age), it may be that the initial loss of synapses between 4 and 12 months simply reflects a regulatory natural pruning (/maturation) process of supernumerary (synaptic) contacts, which are readily observed over the lifetime of all mammals along the developmental continuum. *One similar study also investigated the changes in synaptic density (of presumably excitatory and inhibitory synapses) within the CA3-SL across the life span of F1 Brown Norway X Fisher 344 rats, at 4, 18, and 29 months of age [25]. Although the authors reported a conserved numeric density (of both total and subtypes) of synapses with aging in contrast to our findings, they did not place their data in the context of volumetric changes in the CA3 region.*

A caveat in assessment of these measurements is that volume changes can negate an apparent change in synaptic density because if the tissue volume increases it would confound any true change in synaptic number. Thus ideally one should estimate total synaptic number, rather than density, but to obtain total synaptic number in CA3-SL, it would be necessary to know the volume of this subregion of CA3. This was not feasible in the present study because although it is possible to identify CA3-SL, it is difficult to define boundaries precisely enough to measure its volume alone. However, if we consider the volume of CA3 as a whole, which increases by 24% at 12 months and 33% at 28 months, then a synaptic density decrease of 45% would still indicate a real decrease in total synaptic number. If the percentage values for overall CA3 volume changes are assumed to be similar in CA3-SL and are used theoretically for the purposes of calculating total synapse number in CA3-SL, then there is a 29% decrease in total number from 4 to 12 months and 36% between 4 and 28 months.

In parallel with our previous publication detailing ultrastructural changes in the CA3-SR with ageing (at 22 months) [9], we also report preliminary data showing age-related changes in the neuropil of the CA3-SL at 28 months (and 12 months), typified by the widespread appearance of potentially activated microglia processes, with electron dense spherical fragments resembling intracellular vacuoles, lipofuscin, and phagolysosomes. These glial cell processes were present throughout the CA3-SL neuropil, which is indicative of a greater phagocytic activity and an increased “activation state” at the later age. Unfortunately, due to lack of brain materials at the different time points used in this study (assigned for separate stereological procedures), we were unable to perform further light microscopic/immunohistochemical analysis with appropriate markers of microglial activation such as IBA1 and MHCII, to corroborate our EM finding demonstrating significant microglial activation with ageing. These results are however broadly in keeping with our previous observations in the hippocampal CA3 region [16], including others [26, 27], showing age-related robust changes to microglia (at 2–4, 12, and 22 months of age), accompanied by hypertrophic cell somata, thicker cellular processes, and upregulated cell surface antigen markers of activation (CD11B and MHCII).

Glial cells are known to be primed/activated with advanced ageing [28], prior to any evidence of concurrent major pathology [28]. They feature prominently in most neurodegenerative diseases and are involved in the production of inflammatory cytokines and other cytotoxic compounds. Their age-related altered state or function could thus drive the etiological process seen in aging and especially age-related diseases. From our ultrastructural examination we also observed altered perivascular cell morphology surrounding microvessels with advanced ageing in the CA3. These cells characteristically appeared to express a dense population of lipid droplets and electron dense spherical granules. Perivascular cells such as pericytes, endothelial, astrocytes, and smooth muscle cells form part of the components of the neurovascular unit and play integral roles in providing structural support to the microvasculature, maintaining blood brain barrier (BBB) permeability and cerebral blood flow. The altered morphology of these specific perivascular cells in the CA3 neuropil therefore could imply that there might be microvascular disturbances and possibly alterations in BBB integrity with advanced ageing. Giving the preliminary nature of these finding we believe that further investigations into the significant loss of mossy fibre synapses and the contributions of prominent microglia (and perivascular/microvascular) changes will be vital in understanding the neurobiological processes which occur in the hippocampus with ageing.

## Figures and Tables

**Table 1:** Numerical values of the right dorsal hippocampal volume (top panel); asymmetric synaptic density (middle panel) and quantitative ultrastructural changes to microglia processes (bottom panel) within the CA3-SL with ageing. Total right dorsal hippocampal volume is significantly increased at 28 months of age. Changes are significant in the CA3 only. There is a significant reduction (~43–45%) in the density of mossy fibre asymmetric synapses on CA3 thorny excrescence at 12 and 28 months compared to 4-month-old rats. 12- and 28-month-old animals had a greater number (4-5-fold) of phagolysosome +ve glial processes compared to 4-month-old animals. The number of spherical phagolysosomes per microglial process was increased by 50% at 28 months compared to 12-month-old animals. Values represent mean ± SEM. Asterisks in top panel denote statistical significance (one-way ANOVA) as follows: ∗*P* < 0.05, ∗∗*P* < 0.01, ∗∗∗*P* < 0.001. Synaptic density estimates were determined by one-way ANOVA, based on normalized *N*_*v*_ synaptic values compared to 4-month-old control.

	4 months	12 months	28 months
Right dorsal (anterior) hippocampal volume (mm^3^)			
CA3	2.69 ± 0.07	3.34 ± 0.14∗∗	3.58 ± 0.017∗∗
Total volume	11.64 ± 0.67	12.17 ± 0.08	13.22 ± 0.28
Asymmetric synaptic density (per *μ*m^3^)			
Macular synapses	1.45 ± 0.27	0.80 ± 0.22∗	0.82 ± 0.07∗∗∗
Perforated synapses	0.18 ± 0.02	0.13 ± 0.05	0.08 ± 0.03∗
Total *N*_*v*_ (per *μ*m^3^)	1.63 ± 0.28	0.93 ± 0.26	0.90 ± 0.06∗∗∗
Ultrastructural changes to microglial processes			
Number of phagolysosome +ve glial processes per 100 *μ*m^3^	0.00 ± 0.00	4.21 ± 2.92	5.15 ± 1.23
Number of phagolysosomes per microglial cell process	0.00 ± 0.00	2.6 ± 1.03	5.18 ± 2.00
